# Candidatus List No. 7

**DOI:** 10.1099/ijsem.0.006847

**Published:** 2025-09-01

**Authors:** Marko Kostovski, Aharon Oren, Markus Göker

**Affiliations:** 1 Institute of Microbiology and Parasitology, Faculty of Medicine, University Ss Cyril and Methodius in Skopje, Skopje, Macedonia; 2 The Institute of Life Sciences, The Hebrew University of Jerusalem, The Edmond J. Safra Campus, 9190401 Jerusalem, Israel; 3 Leibniz Institute DSMZ – German Collection of Microorganisms and Cell Cultures, Inhoffenstrasse 7B, Inhoffenstrasse 7B, D-38124 Braunschweig, Germany

## Introduction

Based on the guidelines of Appendix 11 of the International Code of Nomenclature of Prokaryotes (ICNP) [1], a list in the form of a codified record of *Candidatus* names [2,3] should be published at appropriate intervals in the International Journal of Systematic and Evolutionary Microbiology (IJSEM). In order to comply at least partially with the guidelines of Appendix 11, the List Editors have previously presented *Candidatus* List No. 1, which contained an inventory of the names of *Candidatus* taxa compiled from the literature published up to the end of 2018 [4]. *Candidatus* List No. 2, with names proposed in 2019 [5], was published in 2021, and *Candidatus* List Nos. 3 and 4, with names proposed in 2020 and 2021, respectively, were published in 2022 [6,7]. The rank of phylum has recently been included [8] in the rules of the ICNP [1]. The first *Candidatus* Phylum List was published in 2023 [9]; as of *Candidatus* List No. 6 [10], this *Candidatus* Phylum List was considered to be *Candidatus* List No. 5.

While the List Editors continued to search the literature for new *Candidatus* names, another emendation of the ICNP, the addition of Section 10, fundamentally changed the meaning of the IJSEM *Candidatus* lists [11] and superseded Appendix 11 (Rule 66 Note). *Candidatus* names can still not be validly published under the ICNP but can be pro-validly published if certain requirements are met (Rule 67). Inclusion in an IJSEM *Candidatus* list published by 2024 will make a name pro-validly published. From 1 January 2025, publication in the IJSEM, either in an effective publication or in a *Candidatus* list, is a necessary but not a sufficient condition for a name to become pro-validly published, analogous to the conditions for valid publication of a name [1]. Several adjustments to the way *Candidatus* lists are published were therefore necessary [10]. The first list published under the new rules was *Candidatus* List No. 6 [10].

The present list is therefore *Candidatus* List No. 7. It is based on a search of the literature until 2022 for *Candidatus* names with the category order or family that have not yet been included in previous *Candidatus* lists [4,7,9, 10]. If considered suitable for pro-valid publication, the names and those associated with them are included in the main table. The others are listed in appendices 7 and 8. After the first round of assignments of nomenclatural types to taxa with names included in the pre-2025 *Candidatus* lists [Rule 68(2)(a)] was included in List 6 [10], the first set of appendices of the present list proceeds with this work:

- Appendix 1 lists species of *Candidatus* genera that were inadvertently omitted from *Candidatus* Lists 1–4.
- Appendix 2 lists the type species of non-monotypic *Candidatus* genera if designated by the original authors.
- Appendix 3 lists the type species of non-monotypic *Candidatus* genera if none were designated by the original authors, but the List Editors could designate them.
- Appendix 4 lists the type species of *Candidatus* genera to which the original authors did not assign any species, but for which sequence information was provided.
- Appendix 5 lists the *Candidatus* genera to which the original authors did not assign any species and for which they did not provide sequence information.
- Appendix 6 lists type species of *Candidatus* genera that were inadvertently omitted from Appendix 1 of *Candidatus* List No. 6.

The assignment of type species to *Candidatus* genera with names included in the pre-2025 *Candidatus* lists [Rule 68(2)(a)] is thereby supposed to be completed, except for a few cases listed below.

**Table:** 

Name/author	Proposed as	Nomenclatural type^**1**^	Pro-priority^**2**^	Reference
Absconditicoccus praedator Yakimov *et al*. 2022	sp. nov.	M39-6=CP054059	4	[12]
Absconditicoccus Yakimov *et al*. 2022	gen. nov.	Absconditicoccus praedator Yakimov *et al*. 2022	4	[12]
Absconditicoccaceae Yakimov *et al*. 2022	fam. nov.	Absconditicoccus Yakimov *et al*. 2022	4	[12]
Actinochlamydiaceae Steigen *et al*. 2013	fam. nov.	Actinochlamydia Steigen *et al*. 2013	1	[13]
Amarolineaceae Andersen *et al*. 2019	fam. nov.	Amarolinea Andersen *et al*. 2019	2	[14]
Anstonella stagnisolii corrig. Vazquez-Campos *et al*. 2021^3^	sp. nov.	LFW-35=CABMCJ010000000	3	[15]
Anstonella Vazquez-Campos *et al*. 2021	gen. nov.	Anstonella stagnisolii corrig. Vazquez-Campos *et al*. 2021	3	[15]
Anstonellaceae Vazquez-Campos *et al*. 2021	fam. nov.	Anstonella Vazquez-Campos *et al*. 2021	3	[15]
Anstonellales Vazquez-Campos *et al*. 2021	ord. nov.	Anstonella Vazquez-Campos *et al*. 2021	3	[15]
Bilamarchaeum dharawalorum corrig. Vazquez-Campos *et al*. 2021^4^	sp. nov.	LFW-283_2=CABMJJ010000000	3	[15]
Bilamarchaeum Vazquez-Campos *et al*. 2021	gen. nov.	Bilamarchaeum dharawalorum corrig. Vazquez-Campos *et al*. 2021	3	[15]
Bilamarchaeaceae Vazquez-Campos *et al*. 2021	fam. nov.	Bilamarchaeum Vazquez-Campos *et al*. 2021	3	[15]
Burarchaeum australiense Vazquez-Campos *et al*. 2021	sp. nov.	LFW-281_7=CABMJK010000000	3	[15]
Burarchaeum Vazquez-Campos *et al*. 2021	gen. nov.	Burarchaeum australiense Vazquez-Campos *et al*. 2021	3	[15]
Burarchaeaceae Vazquez-Campos *et al*. 2021	fam. nov.	Burarchaeum Vazquez-Campos *et al*. 2021	3	[15]
Burarchaeales Vazquez-Campos *et al*. 2021	ord. nov.	Burarchaeum Vazquez-Campos *et al*. 2021 [15]	3	[15]
Gugararchaeum adminiculibundum corrig. Vazquez-Campos *et al*. 2021^5^	sp. nov.	LFW-121_3=CABMDC000000000	3	[15]
Gugararchaeum Vazquez-Campos *et al*. 2021	gen. nov.	Gugararchaeum adminiculibundum corrig. Vazquez-Campos *et al*. 2021	3	[15]
Gugararchaeaceae Vazquez-Campos *et al*. 2021	fam. nov.	Gugararchaeum Vazquez-Campos *et al*. 2021	3	[15]
Gugararchaeales Vazquez-Campos *et al*. 2021	ord. nov.	Gugararchaeum Vazquez-Campos *et al*. 2021	3	[15]
Nanoanaerosalina halalkaliphila Zhao *et al*. 2022	sp. nov.	NHA20=JALDAE000000000	4	[16]
Nanoanaerosalina Zhao *et al*. 2022	gen. nov.	Nanoanaerosalina halalkaliphila Zhao *et al*. 2022	4	[16]
Nanoanaerosalinaceae Zhao *et al*. 2022	fam. nov.	Nanoanaerosalina Zhao *et al*. 2022	4	[16]
Nanohalalkaliarchaeum halalkaliphilum Zhao *et al*. 2022	sp. nov.	NHA21=JALDAF000000000	4	[16]
Nanohalalkaliarchaeum Zhao *et al*. 2022	gen. nov.	Nanohalalkaliarchaeum halalkaliphilum Zhao *et al*. 2022	4	[16]
Nanohalalkaliarchaeaceae Zhao *et al*. 2022	fam. nov.	Nanohalalkaliarchaeum Zhao *et al*. 2022	4	[16]
Norongarragalina meridionalis Vazquez-Campos *et al*. 2021	sp. nov.	LFW-144_1=CABMDO010000000	3	[15]
Norongarragalina Vazquez-Campos *et al*. 2021	gen. nov.	Norongarragalina meridionalis Vazquez-Campos *et al*. 2021	3	[15]
Norongarragalinaceae Vazquez-Campos *et al*. 2021	fam. nov.	Norongarragalina Vazquez-Campos *et al*. 2021	3	[15]
Norongarragalinales Vazquez-Campos *et al*. 2021	ord. nov.	Norongarragalina Vazquez-Campos *et al*. 2021	3	[15]
Tiddalikarchaeum anstonanum corrig. Vazquez-Campos *et al*. 2021^6^	sp. nov.	LFW-252_1=CABMEV010000000	3	[15]
Tiddalikarchaeum Vazquez-Campos *et al*. 2021	gen. nov.	Tiddalikarchaeum anstonanum corrig. Vazquez-Campos *et al*. 2021	3	[15]
Tiddalikarchaeaceae Vazquez-Campos *et al*. 2021	fam. nov.	Tiddalikarchaeum Vazquez-Campos *et al*. 2021	3	[15]
Tiddalikarchaeales Vazquez-Campos *et al*. 2021	ord. nov.	Tiddalikarchaeum Vazquez-Campos *et al*. 2021	3	[15]

1In the case of a sequence as nomenclatural type, the nucleotide accession number is provided in line with Rule 69.

2Pro-priority number assigned according to the year of the effective publication.

3The List Editors have corrected the epithet from *stagnisolia* to *stagnisolii* (stag.ni.so’li.i. L. neut. n. *stagnum*, a piece of standing water, pool, pond; L. neut. n. *solium*, a tub, bathtub; N.L. gen. n. *stagnisolii*, of overflowing bathtub, in reference to the phenomenon described during heavy rainfalls at the Little Forest Legacy Site trenches).

4The List Editors have corrected the epithet from *dharawalense* to *dharawalorum* (dha.ra.wal.o’rum. N.L. gen. pl. n. *dharawalorum*, pertaining to the Dharawal, traditional owners of the lands where the Little Forest Legacy Site is located).

5The List Editors have corrected the epithet from *adminiculabundum* to *adminiculibundum* (ad.mi.ni.cu.li.bun’dum. L. neut. adj. *adminiculibundum*, self-supporting; in reference to the limited external requirements and its suggested independence from a host/symbiote, due to the predicted presence of pathways for the biosynthesis of amino acids, purines, pyrimidines, thiamine and riboflavin).

6The List Editors have corrected the epithet from *anstoanum* to *anstonanum* [an.ston.a’num. N.L. neut. adj. *anstonanum*, from Australian Nuclear Science and Technology Organisation (ANSTO), institution managing the Little Forest Legacy Site].

## Appendix 1. Species names inadvertently omitted from pre-2025 *Candidatus* lists

Rule 68(2)(a) instructs the List Editors to allocate nomenclatural types to taxa included in pre-2025 *Candidatus* lists, allowing them a certain degree of freedom in doing so. In order to designate type species for genera, some species must first be assigned to these genera. The following table lists genus names included in pre-2025 *Candidatus* lists to which species had been assigned in the effective publication, but whose names were inadvertently omitted from the respective *Candidatus* list. This table serves as a correction to the relevant *Candidatus* list. When determining the date of their pro-valid publication and other nomenclatural aspects, these species names are to be treated as if they had been included in the indicated *Candidatus* list.

**Table:** 

**Genus**	**Species**	***Candidatus*** **list** no.
Neoarthromitus corrig. Snel *et al*. 1995 (=Arthromitus Snel *et al*. 1995)	Neoarthromitus galli corrig. Snel *et al*. 1995 (=Arthromitus galli Snel *et al*. 1995)	1
Neoarthromitus corrig. Snel *et al*. 1995 (=Arthromitus Snel *et al*. 1995)	Neoarthromitus muris corrig. Snel *et al*. 1995 (=Arthromitus muris Snel *et al*. 1995)	1
Neoarthromitus corrig. Snel *et al*. 1995 (=Arthromitus Snel *et al*. 1995)	Neoarthromitus ratti corrig. Snel *et al*. 1995 (=Arthromitus ratti Snel *et al*. 1995)	1
Pinguicoccus Serra *et al*. 2020^1^	Pinguicoccus supinus Serra *et al*. 2020	3

1See also *Candidatus* List No. 6 [10].

## Appendix 2. Designation of type species for non-monotypic genera by their authors

This is the assignment of type species to genera for which more than one species from the same effective publication was included in the pre-2025 *Candidatus* list. Genera that were not monotypic, in the sense that more than one species was proposed or made pro-validly published by inclusion in the same *Candidatus* list, but for which the authors of each publication assigned a type species, are listed below. Inclusion in this table does not imply that the names are pro-legitimate, as there may still be reasons to consider the type species name, and therefore the genus name, as pro-illegitimate. It also does not imply that validly published synonyms or homonyms do not yet exist.

**Table:** 

**Genus**	Type **species**	***Candidatus*** **list** no.
Aceella corrig. Williams *et al.* 2021	Aceella lacicola corrig. Williams *et al*. 2021	4
Acidulidesulfobacter corrig. Tan *et al*. 2019	Acidulidesulfobacter guangdongensis corrig. Tan *et al*. 2019	2
Acidulidesulfobacterium corrig. Tan *et al*. 2019	Acidulidesulfobacterium ferriphilum corrig. Tan *et al*. 2019	2
Allocopromorpha corrig. Gilroy *et al*. 2021	Allocopromorpha excrementavium corrig. Gilroy *et al*. 2021	4
Alloenteromonas corrig. Gilroy *et al*. 2021	Alloenteromonas pullistercoris corrig. Gilroy *et al*. 2021	4
Aphodousia Gilroy *et al*. 2021	Aphodousia faecavium Gilroy *et al*. 2021	4
Aphodovivens Gilroy *et al*. 2021	Aphodovivens avicola Gilroy *et al*. 2021	4
Aveggerthella Gilroy *et al*. 2021	Aveggerthella excrementigallinarum Gilroy *et al*. 2021	4
Avibacteroides Gilroy *et al*. 2021	Avibacteroides excrementipullorum Gilroy *et al*. 2021	4
Avilachnospira Gilroy *et al*. 2021	Avilachnospira avistercoris Gilroy *et al*. 2021	4
Avimicrobium Glendinning *et al*. 2021	Avimicrobium caecorum Glendinning *et al*. 2021	4
Avimonas Glendinning *et al*. 2021	Avimonas caecicola Glendinning *et al*. 2021	4
Avisuccinivibrio Gilroy *et al*. 2021	Avisuccinivibrio stercorigallinarum Gilroy *et al*. 2021	4
Avoscillospira Gilroy *et al*. 2021	Avoscillospira stercorigallinarum Gilroy *et al*. 2021	4
Caccocola Gilroy *et al*. 2021	Caccocola faecigallinarum Gilroy *et al*. 2021	4
Caccoplasma Gilroy *et al*. 2021	Caccoplasma merdavium Gilroy *et al*. 2021	4
Caccousia Gilroy *et al*. 2021	Caccousia avicola Gilroy *et al*. 2021	4
Celaenobacter Williams *et al*. 2021	Celaenobacter antarcticus Williams *et al*. 2021	4
Choladousia Gilroy *et al*. 2021	Choladousia intestinavium Gilroy *et al*. 2021	4
Coprenecus Gilroy *et al*. 2021	Coprenecus pullicola Gilroy *et al*. 2021	4
Copromonas Gilroy *et al*. 2021	Copromonas avistercoris Gilroy *et al*. 2021	4
Coproplasma Gilroy *et al*. 2021	Coproplasma stercoravium Gilroy *et al*. 2021	4
Cryosericum Martinez *et al*. 2019	Cryosericum septentrionale Martinez *et al*. 2019	2
Cryptobacteroides Gilroy *et al*. 2021	Cryptobacteroides avicola Gilroy *et al*. 2021	4
Egerieicola Gilroy *et al*. 2021	Egerieicola faecalis Gilroy *et al*. 2021	4
Egerieimonas Gilroy *et al*. 2021	Egerieimonas intestinavium Gilroy *et al*. 2021	4
Enterenecus Gilroy *et al*. 2021	Enterenecus merdae Gilroy *et al*. 2021	4
Enterousia Gilroy *et al*. 2021	Enterousia excrementavium Gilroy *et al*. 2021	4
Faecimonas Gilroy *et al*. 2021	Faecimonas intestinavium Gilroy *et al*. 2021	4
Faeciplasma Gilroy *et al*. 2021	Faeciplasma avium Gilroy *et al*. 2021	4
Faecivivens Gilroy *et al*. 2021	Faecivivens stercorigallinarum Gilroy *et al*. 2021	4
Faecousia Gilroy *et al*. 2021	Faecousia intestinigallinarum Gilroy *et al*. 2021	4
Fimenecus Gilroy *et al*. 2021	Fimenecus excrementigallinarum Gilroy *et al*. 2021	4
Fimicola Gilroy *et al*. 2021	Fimicola merdigallinarum Gilroy *et al*. 2021	4
Fimimonas Gilroy *et al*. 2021	Fimimonas gallinarum Gilroy *et al*. 2021	4
Fimimorpha Gilroy *et al*. 2021	Fimimorpha faecalis Gilroy *et al*. 2021	4
Finniella Hess *et al*. 2016	Finniella lucida Hess *et al*. 2016	1
Gallacutalibacter Gilroy *et al*. 2021	Gallacutalibacter pullicola Gilroy *et al*. 2021	4
Galligastranaerophilus Gilroy *et al*. 2021	Galligastranaerophilus faecipullorum Gilroy *et al*. 2021	4
Gallimonas Glendinning *et al*. 2021	Gallimonas caecicola Glendinning *et al*. 2021	4
Galloscillospira Gilroy *et al*. 2021	Galloscillospira excrementipullorum Gilroy *et al*. 2021	4
Halomarinus Savoie *et al*. 2021	Halomarinus kaneohensis Savoie *et al*. 2021	4
Heritagella Glendinning *et al*. 2021	Heritagella caecorum Glendinning *et al*. 2021	4
Kaelpia Williams *et al*. 2021	Kaelpia aquatica Williams *et al*. 2021	4
Limadaptatus Gilroy *et al*. 2021	Limadaptatus stercoripullorum Gilroy *et al*. 2021	4
Limiplasma Gilroy *et al*. 2021	Limiplasma pullicola Gilroy *et al*. 2021	4
Limisoma Gilroy *et al*. 2021	Limisoma faecipullorum Gilroy *et al*. 2021	4
Limivivens Gilroy *et al*. 2021	Limivivens intestinipullorum Gilroy *et al*. 2021	4
Merdimorpha Gilroy *et al*. 2021	Merdimorpha intestinavium Gilroy *et al*. 2021	4
Merdisoma Gilroy *et al*. 2021	Merdisoma merdipullorum Gilroy *et al*. 2021	4
Merdivicinus Gilroy *et al*. 2021	Merdivicinus faecavium Gilroy *et al*. 2021	4
Merdivivens Gilroy *et al*. 2021	Merdivivens pullistercoris Gilroy *et al*. 2021	4
Metaruminococcus Glendinning *et al*. 2021	Metaruminococcus caecorum Glendinning *et al*. 2021	4
Neoanaerotignum Glendinning *et al*. 2021	Neoanaerotignum galli Glendinning *et al*. 2021	4
Onthocola Gilroy *et al*. 2021	Onthocola gallistercoris Gilroy *et al*. 2021	4
Onthoplasma Gilroy *et al*. 2021	Onthoplasma faecipullorum Gilroy *et al*. 2021	4
Onthousia Gilroy *et al*. 2021	Onthousia faecavium Gilroy *et al*. 2021	4
Ornithoclostridium Gilroy *et al*. 2021	Ornithoclostridium excrementipullorum Gilroy *et al*. 2021	4
Ornithomonoglobus Gilroy *et al*. 2021	Ornithomonoglobus merdipullorum Gilroy *et al*. 2021	4
Ornithospirochaeta Gilroy *et al*. 2021	Ornithospirochaeta stercoravium Gilroy *et al*. 2021	4
Paralachnospira Glendinning *et al*. 2021	Paralachnospira avium Glendinning *et al*. 2021	4
Phytoplasma Firrao *et al*. 2004^1^	Phytoplasma asteris Lee *et al*. in Firrao *et al*. 2004	1
Protoclostridium Glendinning *et al*. 2021	Protoclostridium gallicola Glendinning *et al*. 2021	4
Pseudoscillospira corrig. Glendinning *et al*. 2021	Pseudoscillospira faecavium corrig. Glendinning *et al*. 2021	4
Pullichristensenella Gilroy *et al*. 2021	Pullichristensenella avicola Gilroy *et al*. 2021	4
Pullilachnospira Gilroy *et al*. 2021	Pullilachnospira stercoravium Gilroy *et al*. 2021	4
Scatocola Gilroy *et al*. 2021	Scatocola faecipullorum Gilroy *et al*. 2021	4
Scatomonas Gilroy *et al*. 2021	Scatomonas merdigallinarum Gilroy *et al*. 2021	4
Scatomorpha Gilroy *et al*. 2021	Scatomorpha merdavium Gilroy *et al*. 2021	4
Scatosoma Gilroy *et al*. 2021	Scatosoma pullicola Gilroy *et al*. 2021	4
Scatousia Gilroy *et al*. 2021	Scatousia excrementipullorum Gilroy *et al*. 2021	4
Scybalocola Gilroy *et al*. 2021	Scybalocola faecigallinarum Gilroy *et al*. 2021	4
Scybalomonas Gilroy *et al*. 2021	Scybalomonas excrementigallinarum Gilroy *et al*. 2021	4
Stercoripulliclostridium Gilroy *et al*. 2021	Stercoripulliclostridium merdipullorum Gilroy *et al*. 2021	4
Stygiibacter corrig. Williams *et al*. 2021	Stygiibacter australis corrig. Williams *et al*. 2021	4
Tenebribacter Williams *et al*. 2021	Tenebribacter burtonii Williams *et al*. 2021	4
Termititenax Utami *et al*. 2019	Termititenax aidoneus Utami *et al*. 2019	2
Ventrenecus Gilroy *et al*. 2021	Ventrenecus avicola Gilroy *et al*. 2021	4
Ventricola Gilroy *et al*. 2021	Ventricola intestinavium Gilroy *et al*. 2021	4

1Although the authors did not explicitly designate a type species for the genus, they provided the same reference sequence, M30790, for *Candidatus* Phytoplasma Firrao *et al.* 2004 and for *Candidatus* Phytoplasma asteris Lee *et al.* in Firrao *et al.* 2004.

## Appendix 3. Designation of type species for non-monotypic genera by the List Editors

This table shows the type species assigned by the List Editors to genera that included more than one species from the same effective publication in the pre-2025 *Candidatus* list. The genera that were not monotypic – meaning that more than one species was proposed or made validly published by inclusion in the same *Candidatus* list – and for which the authors of each publication did not assign a type species are listed below. The List Editors have chosen one of these species as the type species (lectotype). Due consideration was given as to whether nucleotide sequences were available for the respective species. If this criterion made no difference, the species that was either first described or first mentioned in the effective publication was chosen. Inclusion in this table does not imply that the names are pro-legitimate, as there may still be reasons to consider the type species name, and therefore the genus name, as pro-illegitimate. It also does not imply that validly published synonyms or homonyms do not yet exist.

**Table:** 

**Genus**	Type **species**	***Candidatus*** **list** no.
Abditibacter Grieb *et al*. 2020	Abditibacter vernus Grieb *et al*. 2020	3
Acidiferrum corrig. Epihov *et al*. 2021	Acidiferrum typicum corrig. Epihov *et al*. 2021	4
Berkiella Mehari *et al*. 2016	Berkiella aquae Mehari *et al*. 2016	1
Blochmanniella corrig. Sauer *et al*. 2000	Blochmanniella floridana corrig. Sauer *et al*. 2000	1
Caldatribacterium Dodsworth *et al*. 2013	Caldatribacterium californiense corrig. Dodsworth *et al*. 2013	1
Cybelea Ji *et al*. 2021	Cybelea septentrionalis Ji *et al*. 2021	4
Cytomitobacter Tashyreva *et al*. 2018	Cytomitobacter primus Tashyreva *et al*. 2018	2
Elarobacter Ji *et al*. 2021	Elarobacter winogradskyi Ji *et al*. 2021	4
Electronema Trojan *et al*. 2016	Electronema nielsenii Trojan *et al*. 2016	1
Electrothrix Trojan *et al*. 2016	Electrothrix marina Trojan *et al*. 2016	1
Endomicrobium Stingl *et al*. 2005	Endomicrobium trichonymphae Stingl *et al*. 2005	1
Endowatersipora Anderson and Haygood 2007	Endowatersipora glebosa corrig. Anderson and Haygood 2007	1
Epulonipiscioides corrig. Ngugi *et al*. 2017	Epulonipiscioides gigas corrig. Ngugi *et al*. 2017	1
Fritschea Everett *et al*. 2005	Fritschea bemisiae Everett *et al*. 2005	1
Gigantothauma corrig. Muller *et al*. 2010	Gigantothauma karukerense corrig. Muller *et al*. 2010	1
Ichthyocystis Seth-Smith *et al*. 2016	Ichthyocystis spari corrig. Seth-Smith *et al*. 2016	4
Kinetoplastidibacterium corrig. Du *et al*. 1994	Kinetoplastidibacterium blastocrithidiae corrig. Du *et al*. 1994	1
Liberibacter corrig. Jagoueix *et al*. 1994	Liberibacter asiaticus corrig. Jagoueix *et al*. 1994	1
Liberibacter corrig. Jagoueix *et al*. 1994	Liberibacter asiaticus corrig. Murray and Stackebrandt 1995	1
Lustribacter Ji *et al*. 2021	Lustribacter caenicola Ji *et al*. 2021	4
Macropleicola Kölsch *et al*. 2009	Macropleicola chrysomelidarum corrig. Kölsch *et al*. 2009	1
Magnetananas Chen *et al*. 2015	Magnetananas tsingtaonensis corrig. Chen *et al*. 2015	1
Methanomethylicus Vanwonterghem *et al*. 2016	Methanomethylicus mesodigestus corrig. Vanwonterghem *et al*. 2016	1
Methanomixotrophus corrig. Liu *et al*. 2020	Methanomixotrophus dualis corrig. Liu *et al*. 2020	3
Methylopumilus Salcher *et al*. 2015	Methylopumilus planktonicus Salcher *et al*. 2015	1
Nanopelagicus Neuenschwander *et al*. 2018	Nanopelagicus limnae corrig. Neuenschwander *et al*. 2018	1
Nanosyncoccus McLean *et al*. 2020	Nanosyncoccus alcis corrig. McLean *et al*. 2020	3
Neoarthromitus corrig. Snel *et al*. 1995	Neoarthromitus galli corrig. Snel *et al*. 19951	1
Nitrosodeserticola Hwang *et al*. 2021	Nitrosodeserticola atacamensis corrig. Hwang *et al*. 2021	4
Nucleicoccus corrig. Sato *et al*. 2014	Nucleicoccus trichonymphae corrig. Sato *et al*. 2014	1
Paracaedibacter Horn *et al*. 1999	Paracaedibacter acanthamoebae Horn *et al*. 1999	1
Parvarchaeum Baker *et al*. 2010	Parvarchaeum acidiphilum Baker *et al*. 2010	1
Pleuronema corrig. Couradeau *et al*. 2017	Pleuronema perforans corrig. Couradeau *et al*. 2017	3
Profftia Toenshoff *et al*. 2012	Profftia tarda Toenshoff *et al*. 2012	1
Rhodoluna Hahn 2009	Rhodoluna lacicola Hahn 2009	1
Scalindua Schmid *et al*. 2003	Scalindua brodae Schmid *et al*. 2003	1
Sifarchaeum corrig. Farag *et al*. 2021	Sifarchaeum marinarchaeum corrig. Farag *et al*. 2021	4
Syntropharchaeum corrig. Laso-Pérez *et al*. 2016	Syntropharchaeum butanivorans corrig. Laso-Pérez *et al*. 2016	1
Thalassarchaea corrig. Martin-Cuadrado *et al*. 2015	Thalassarchaea mediterranei corrig. Martin-Cuadrado *et al*. 2015	1
Thiobarba Assié *et al*. 2020	Thiobarba azorica corrig. Assié *et al*. 2020	3
Vallotiella corrig. Toenshoff *et al*. 2012	Vallotiella hemipterorum corrig. Toenshoff *et al*. 2012	1
Xenobium Ji *et al*. 2021	Xenobium occultum Ji *et al*. 2021	4
Xiphinematobacter Vandekerckhove *et al*. 2000	Xiphinematobacter longidoridarum corrig. Vandekerckhove *et al*. 2000	1

1See Appendix 1.

## Appendix 4. Species names for genera lacking species but with sequence information

The formation of the earliest *Candidatus* names shows an anomaly. The original proposal for the use of these names [2] stipulated that the word ‘*Candidatus*’ should be ‘followed by a descriptive vernacular epithet’. This resulted in the names ‘*Candidatus* intracellularis’ and ‘*Candidatus* magnetobacterium’ [2]. However, Murray and Stackebrandt [3] later also listed the names ‘*Candidatus* Liberobacter asiaticum’ and ‘*Candidatus* Liberobacter africanum’, in which the word ‘*Candidatus*’ was immediately followed by a genus name rather than an epithet. In line with Appendix 11 of the ICNP [1], the *Candidatus* List No. 1 [4] interpreted ‘intracellularis’ and ‘magnetobacterium’ in the above context as genus names. However, in such situations, species epithets would need to be found for these genera. Section 10 of the ICNP [11] also expects *Candidatus* genera to harbour species.

Nevertheless, *Candidatus* List Nos. 1–4 included the names of several genera to which the effective publication did not assign any species. The List Editors assessed these names and found that, for the majority, the effective publication provided sequence information that was either given directly as INSDC nucleotide accession numbers, or given as other identifiers that could be mapped to such accession numbers, as required by Section 10 of the ICNP [11]. The purpose of treating *Candidatus* names included in the pre-2025 *Candidatus* lists as pro-validly published is to stabilize the nomenclature of these names, which are often well known [17]. For this reason, Section 10 of the ICNP gives the List Editors considerable freedom in determining the nomenclatural types of the included taxa [11]. The List Editors have therefore chosen to assign new species names to pro-validly published *Candidatus* genus names lacking species, but associated with appropriate DNA sequence information. These sequence data are then assigned as nomenclatural types of the proposed species names.

To this end, the neutral species epithets L. masc. adj. *primus*, L. fem. adj. *prima* or L. neut. adj. *primum*, meaning ‘first’, were chosen. The following table serves as a correction to the relevant *Candidatus* list. When determining the date of their pro-valid publication and other nomenclatural aspects, these species names should be treated as if they had been included in the indicated *Candidatus* list. To determine the date of their pro-valid publication and other nomenclatural aspects, these species names should be treated as if they had been included in the indicated *Candidatus* list and the effective publication cited within it. Inclusion in this table does not imply that the names are pro-legitimate, as there may still be reasons to consider the genus name as pro-illegitimate. It also does not imply that validly published synonyms or homonyms do not yet exist.

**Table:** 

**Genus**	***Candidatus*** **list** no.	Type **species**	Nomenclatural type of the **species**
Alcium Solden *et al*. 2017	1	Alcium primum Solden *et al*. 2017	F082=LPNG01000000
Bacilliplasma corrig. Kostanjšek *et al*. 2007	1	Bacilliplasma primum Kostanjšek *et al*. 2007	P5=DQ485974
Bathoplasma corrig. Zhu *et al*. 2020	3	Bathoplasma primum Zhu *et al*. 2020	NZ=JABENI010000000
Bathosphaera Mehrshad *et al*. 2018	2	Bathosphaera prima Mehrshad *et al*. 2018	RH-chloro-G1=QWQU00000000
Caldipriscus Colman *et al*. 2016	2	Caldipriscus primus Colman *et al*. 2016	T1.1=LBFP01000000
Changshengia Woodcroft *et al*. 2018	3	Changshengia prima Woodcroft *et al*. 2018	bog_835=PMPA01000000
Comitans Jacobi *et al*. 1996	1	Comitans primus Jacobi *et al*. 1996	CJ3=X91814
Defluviifilum Nierychlo *et al*. 2019	2	Defluviifilum primum Nierychlo *et al*. 2019	WBA-3=JQ180412
Epifloribacter corrig. Xia *et al*. 2008	1	Epifloribacter primus Xia *et al*. 2008	Epr 91=EF523467
Frackibacter Booker *et al*. 2017	1	Frackibacter primus Booker *et al*. 2017	WG11=FMZT01000000
Haloredivivus Ghai *et al*. 2011	1	Haloredivivus primus Ghai *et al*. 2011	G17=AGNT01000000
Hemicellulosilyticus corrig. Solden *et al*. 2017	1	Hemicellulosilyticus primus Solden *et al*. 2017	F083=LPNF01000000
Izemoplasma corrig. Skennerton *et al*. 2016	1	Izemoplasma primum Skennerton *et al*. 2016	HR1=CP009415
Limnocylindrus Mehrshad *et al*. 2018	2	Limnocylindrus primus Mehrshad *et al*. 2018	ZSMar2m-chloro-G89=QWRJ01000000
Lokiarchaeum corrig. Spang *et al*. 2015	1	Lokiarchaeum primum Spang *et al*. 2015	GC14_75=JYIM01000000
Lumbricincola Nechitaylo *et al*. 2009	1	Lumbricincola primus Nechitaylo *et al*. 2009	Lt-A1=FM165583
Marcellius Nixon *et al*. 2019	2	Marcellius primus Nixon *et al*. 2019	5H=WOUP01000000
Mariniplasma corrig. Mortzfeld *et al*. 2016	3	Mariniplasma primum Mortzfeld *et al*. 2016	Nvec1=KR973435
Nanopetraeus corrig. Crits-Christoph *et al*. 2016	1	Nanopetraeus primus Crits-Christoph *et al*. 2016	SG9=CP012986
Nanosalina Narasingarao *et al*. 2012	1	Nanosalina prima Narasingarao *et al*. 2012	J07AB43=AEIY01000000
Nanosalinicola corrig. Narasingarao *et al*. 2012	1	Nanosalinicola primus Narasingarao *et al*. 2012	J07AB56=AEIX01000000
Nanosynsaccharibacterium corrig. McLean *et al*. 2020	3	Nanosynsaccharibacterium primum McLean *et al*. 2020	TM7_ANC_38.39_G1_1=PQNZ01000000
Nardonella Lefèvre *et al*. 2004	1	Nardonella prima Lefèvre *et al*. 2004	Endosymbiont of Yuccaborus frontalis=AY262022
Nitromaritima Ngugi *et al*. 2016	1	Nitromaritima prima Ngugi *et al*. 2016	SCGC AAA799-A02=JZKI01000000
Parepulonipiscium corrig. Ngugi *et al*. 2017	1	Parepulonipiscium primum Ngugi *et al*. 2017	PG-Nele67M_MBin01=LNZO01000000
Poriferisulfidus corrig. Lavy *et al*. 2018	1	Poriferisulfidus primus Lavy *et al*. 2018	TsSOB=MRSX01000000
Profundisolitarius Mehrshad *et al*. 2018	2	Profundisolitarius primus Mehrshad *et al*. 2018	ZSMay80m-chloro-G1=QWRL01000000
Rubrimentiphilum Sheremet *et al*. 2020	4	Rubrimentiphilum primum Sheremet *et al*. 2020	bin6A=JAAXCW010000000
Salinibacter Antón *et al*. 2000	1	Salinibacter primus Antón *et al*. 2000	EHB-1=AJ133744
Sarcinithrix Nierychlo *et al*. 2019	2	Sarcinithrix prima Nierychlo *et al*. 2019	RS-B44=KC541098
Sulfuripaludibacter corrig. Hausmann *et al*. 2018	1	Sulfuripaludibacter primus Hausmann *et al*. 2018	SbA3=MG182084
Symbiopectobacterium Martinson *et al*. 2020	3	Symbiopectobacterium primum Martinson *et al*. 2020	SyHa=PEHF01000000
Termiticorpusculum Loh *et al*. 2021	4	Termiticorpusculum primum Loh *et al*. 2021	Co191P1bin46=WQRU01000000
Termitimicrobium Loh *et al*. 2021	4	Termitimicrobium primum Loh *et al*. 2021	Emb289P1bin127=WQVG01000000
Thermoproauctor Colman *et al*. 2016	2	Thermoproauctor primus Colman *et al*. 2016	T2.1=LBFR01000000
Thiosymbium corrig. Zimmermann *et al*. 2016	1	Thiosymbium primum Zimmermann *et al*. 2016	XYBE8228=KP943984
Umbricyclops Mehrshad *et al*. 2018	2	Umbricyclops primus Mehrshad *et al*. 2018	ZSMay80m-chloro-G79=QWRN01000000
Villigracilis Nierychlo *et al*. 2019	2	Villigracilis prima Nierychlo *et al*. 2019	Q7358-HYSA=JN391853

## Appendix 5. Species names for genera lacking species and sequence information

A low number of *Candidatus* genera included in *Candidatus* Lists 1–4 lacked both species and sequence information. In four cases, sequence identifiers were provided, but these could not be mapped to INSDC nucleotide accession numbers, as required by Section 10 of the ICNP [11]. These cases will remain open until the List Editors have completed the assignment of the other nomenclatural types. Authors are encouraged to provide the missing information in advance. In one instance, no sequence identifiers were provided, but a species name assigned to the same genus was included in a later pre-2025 *Candidatus* list. This species was chosen as the lectotype.

**Table:** 

**Genus**	***Candidatus*** **list** no.	Type **species**
Dormiibacter corrig. Ji *et al*. 2017	2	Pending
Dwaynesavagella corrig. Thompson *et al*. 2012	1	Dwaynesavagella gallinarum corrig. Gilroy *et al*. 2021 (*Candidatus* List No. 4)
Ferristratum McAllister *et al*. 2021	4	Pending
Ferrooxidibacter corrig. Bethencourt *et al*. 2020	3	Pending
Tianyaibacterium corrig. Cui *et al*. 2021	4	Pending

## Appendix 6. Assignment of type species to monotypic genera

This is the assignment of type species to genera for which only a single species, or only a single species from the same effective publication, was included in the same pre-2025 *Candidatus* list. Almost all of this work was conducted in Appendix 1 of *Candidatus* List No. 6. The following table only includes the names that were inadvertently omitted from that appendix.

**Table:** 

**Genus**	**Species**	*Candidatus* list no.
Nanoperiodontomorbus corrig. McLean *et al*. 2020^1^	Nanoperiodontomorbus periodonticus corrig. McLean et al. 2020	3

1Name inadvertently omitted from Appendix 1 of *Candidatus* List No. 6 because *Candidatus* List No. 3 did not list the species with the corrected spelling of the genus name.

## Appendix 7. Other names at the rank of order

List of *Candidatus* order names proposed up to 2022 that have not yet been included in a *Candidatus* list. These names are not considered to be pro-validly published by virtue of their inclusion in this publication. Comments indicate why we doubt that they can become pro-validly published based on the given effective publication. The names are listed here solely to provide such comments, to alert readers to the existence of these names and to provide spelling corrections. Abbreviations used are as follows: D!, no description given (at least not in main document), including incidental mentions (without actually proposing the name); E!, no etymology provided; F!, name not formed from name of nomenclatural type; H!, type is a homonym of a validly published name; S!, INSDC nucleotide accessions not found for the type species of the type genus, or description of type genus provided in supplement; and T!, no nomenclatural type designated (at least not in main document).

**Table:** 

Proposed name of the ***Candidatus*** taxon^**1**^	Etymology	Nomenclatural type	Reference	Comment
Absconditibacterales (=Absconditabacterales)	We propose correcting the name to Absconditibacterales (Ab.scon.di.ti.bac.te.ra’les. N.L. masc. n. Absconditibacter, a yet-to-be-proposed *Candidatus* genus name; *-ales*, ending to denote an order; N.L. fem. pl. n. Absconditibacterales, the *Candidatus* Absconditibacter order)		Yakimov *et al.* 2022 [12]	D!; T!
Alkanophagales	Al.ka.no.pha.ga’les. N.L. fem. n. Alkanophaga, a yet-to-be-proposed *Candidatus* genus name; *-ales*, ending to denote an order; N.L. fem. pl. n. Alkanophagales, the *Candidatus* Alkanophaga order		Wang *et al.* 2021 [18]	D!; T!
Aminicenantales	A.mi.ni.ce.nan.ta’les. N.L. masc. n. Aminicenans, a *Candidatus* genus name; *-ales*, ending to denote an order; N.L. fem. pl. n. Aminicenantales, the *Candidatus* Aminicenans order	Aminicenans Rinke *et al*. 2013 [19]	Kadnikov *et al.* 2019 [20]	D!
Babelales (=Babeliales)	We propose correcting the name to Babelales (Ba.be.la’les. N.L. masc. n. Babela, a *Candidatus* genus name; *-ales*, ending to denote an order; N.L. fem. pl. n. Babelales, the *Candidatus* Babela order)	Babela Cohen *et al*. 2011 [21]	Yeoh *et al.* 2016 [22]	D!
Comantemaeales (=Comantemales)	We propose correcting the name to Comantemaeales (Co.man.te.mae.a’les. N.L. fem. n. Comantemaea, a yet-to-be-proposed *Candidatus* genus; *-ales*, ending to denote an order; N.L. fem. pl. n. Comantemaeales, the *Candidatus* Comantemaea order)	Borkfalki Hildebrand *et al*. 2019 [23]	Hildebrand *et al*. 2019 [23]	F!; synonym of *Candidatus* Borkfalkiales Hildebrand *et al*. 2020
Cenarchaeales	Cen.ar.chae.a’les. N.L. neut. n. Cenarchaeum, a *Candidatus* genus name; *-ales*, ending to denote an order; N.L. fem. pl. n. Cenarchaeales, the *Candidatus* Cenarchaeum order	Cenarchaeum DeLong and Preston 1996 [24]	DeLong and Preston 1996 [24]	E!
Deferrimicrobiales	De.fer.ri.mi.cro.bi.a’les. N.L. neut. n. Deferrimicrobium, a *Candidatus* genus name; *-ales*, ending to denote an order; N.L. fem. pl. n. Deferrimicrobiales, the *Candidatus* Deferrimicrobium order	Deferrimicrobium Begmatov *et al*. 2022 [25]	Begmatov *et al*. 2022 [25]	D!
Hadarchaeales	Had.ar.chae.a’les. N.L. neut. n. Hadarchaeum, a *Candidatus* genus name; *-ales*, ending to denote an order; N.L. fem. pl. n. Hadarchaeales, the *Candidatus* Hadarchaeum order	Hadarchaeum Chuvochina *et al*. 2019 [26]	Chuvochina *et al*. 2019 [26]	D!
Hadesarchaeales	Ha.des.ar.chae.a’les. N.L. neut. n. Hadesarchaeum, a *Candidatus* genus name; *-ales*, ending to denote an order; N.L. fem. pl. n. Hadesarchaeales, the *Candidatus* Hadesarchaeum order	Hadesarchaeum Hua *et al*. 2019 [27]	Hua *et al*. 2019 [27]	D!
Haloplasmatales	Ha.lo.plas.ma.ta’les. N.L. neut. n. Haloplasma, a *Candidatus* genus name; *-ales*, ending to denote an order; N.L. fem. pl. n. Haloplasmatales, the *Candidatus* Haloplasma order	Haloplasma Zhou *et al*. 2022 [28] (*non Haloplasma* Antunes *et al*. 2008 [29])	Zhou *et al*. 2022 [28]	D!; H!
Heimdallarchaeales	Heim.dall.ar.chae.a’les. N.L. neut. n. Heimdallarchaeum, a *Candidatus* genus name; *-ales*, ending to denote an order; N.L. fem. pl. n. Heimdallarchaeales, the *Candidatus* Heimdallarchaeum order	Heimdallarchaeum Spang *et al*. 2019 [30]	Wu *et al.* 2022 [31]	D!
Hydrothermales	Hy.dro.ther.ma’les. N.L. masc. n. Hydrothermus, a *Candidatus* genus name; *-ales*, ending to denote an order; N.L. fem. pl. n. Hydrothermales, the *Candidatus* Hydrothermus order	Hydrothermus Chuvochina *et al*. 2019 [26]	Chuvochina *et al*. 2019 [26]	D!
Hydrothermarchaeales	Hy.dro.therm.ar.chae.a’les. N.L. neut. n. Hydrothermarchaeum, a *Candidatus* genus name; *-ales*, ending to denote an order; N.L. fem. pl. n. Hydrothermarchaeales, the *Candidatus* Hydrothermarchaeum order	Hydrothermarchaeum Chuvochina *et al*. 2019 [26]	Chuvochina *et al*. 2019 [26]	D!
Izemoplasmatales (=Izemoplasmales)	We propose correcting the name to Izemoplasmatales (I.ze.mo.plas.ma.ta’les. N.L. neut. n. Izemoplasma, a *Candidatus* genus name; *-ales*, ending to denote an order; N.L. fem. pl. n. Izemoplasmatales, the *Candidatus* Izemoplasma order)	Izemoplasma Skennerton *et al*. 2016 [32]	Zheng *et al.* 2021 [33]	D!
Limitiplasmatales (=Yaplasmales)	We propose correcting the name to Limitiplasmatales (Li.mi.ti.plas.ma.ta’les. N.L. neut. n. Limitiplasma, a yet-to-be-proposed *Candidatus* genus name; L. fem. pl. n. suff. *-ales*, ending to denote an order; N.L. fem. pl. n. Limitiplasmatales, the *Candidatus* Limitiplasma order)		Zheng *et al.* 2022 [34]	D!; T!
Luniplasmatales (=Lunaplasmatales)	We propose correcting the name to Luniplasmatales (Lu.ni.plas.ma.ta’les N.L. neut. n. Luniplasma, a *Candidatus* genus name; *-ales*, ending to denote an order; N.L. pl. n. Luniplasmatales, the *Candidatus* Luniplasma order)	Lunaplasma Zinke *et al*. 2021 [35]	Zinke *et al*. 2021 [35]	D!
Methanomethylarchaeales (=Methanomethyloarchaeales)	We propose correcting the name to Methanomethylarchaeales (Me.tha.no.me.thyl.ar.chae.a’les. N.L. neut. n. Methanomethylarchaeum, a *Candidatus* genus name; *-ales*, ending to denote an order; N.L. fem. pl. n. Methanomethylarchaeales, the *Candidatus* Methanomethylarchaeum order)	Methanomethyloarchaeum Hua *et al*. 2019 [27]	Hua *et al*. 2019 [27]	D!
Methanomethylovorales	Me.tha.no.me.thy.lo.vo.ra’les. N.L. masc. n. Methanomethylovorus, a *Candidatus* genus name; *-ales*, ending to denote an order; N.L. fem. pl. n. Methanomethylovorales, the *Candidatus* Methanomethylovorus order	Methanomethylovorus Hua *et al*. 2019 [27]	Hua *et al*. 2019 [27]	D!
Methylacidiphilales	Me.thyl.a.ci.di.phi.la’les. N.L. masc. n. Methylacidiphilum, a *Candidatus* genus name; *-ales*, ending to denote an order; N.L. fem. pl. n. Methylacidiphilales, the *Candidatus* Methylacidiphilales order	Methylacidiphilum Hou *et al*. 2008 [36]	Op den Camp *et al.* 2009 [37]	D!
Methylarchaeales	Me.thyl.ar.chae.a’les. N.L. neut. n. Methylarchaeum, a *Candidatus* genus name; *-ales*, ending denoting an order; N.L. fem. pl. n. Methylarchaeales, the *Candidatus* Methylarchaeum order	Methylarchaeum Hua *et al*. 2019 [27]	Ou *et al.* 2022 [38]	D!
Monstrimarinales (=Monstramariales)	We propose correcting the name to Monstrimarinales (Mon.stri.ma.ri.na’les. N.L. fem. n. Monstrimarina, a yet-to-be-proposed *Candidatus* genus name; *-ales*, ending to denote an order; N.L. fem. pl. n. Monstrimarinales, the *Candidatus* Monstrimarina order)		Landry *et al.* 2017 [39]	D!; T!
Nanosynbacterales	Na.no.syn.bac.te.ra’les. N.L. masc. n. Nanosynbacter, a *Candidatus* genus name; *-ales*, ending to denote an order; N.L. fem. pl. n. Nanosynbacterales, the *Candidatus* Nanosynbacter order	Nanosynbacter McLean *et al*. 2020 [40]	McLean *et al.* 2020 [40]	D!
Nanohydrothermales	Na.no.hy.dro.ther.ma’les. N.L. masc. n. Nanohydrothermus, a *Candidatus* genus name; L. fem. pl. suff. *-ales*, ending to denote an order; N.L. fem. pl. n. Nanohydrothermales, the *Candidatus* Nanohydrothermus order	Nanohydrothermus Xie *et al*. 2022 [41]	Xie *et al.* 2022 [41]	D!; S!
Nucleotidisoterales	Nu.cle.o.ti.di.so.ter.a’les N.L. masc. n. Nucleotidisoter, a *Candidatus* genus name; L. fem. pl. suff. *-ales*, ending to denote an order; N.L. fem. pl. n. Nucleotidisoterales, the *Candidatus* Nucleotidisoter order	Nucleotidisoter Xie *et al*. 2022 [41]	Xie *et al.* 2022 [41]	D!; S!
Obscuribacterales	Ob.scu.ri.bac.te.ra’les. N.L. masc. n. Obscuribacter, a *Candidatus* genus name; *-ales*, ending to denote an order; N.L. fem. pl. n. Obscuribacterales, the *Candidatus* Obscuribacter order	Obscuribacter Soo *et al*. 2014 [42]	Xie *et al.* 2022 [42]	D!
Procaibacterales (=Procabacteriales)	We propose correcting the name to Procaibacterales (Pro.ca.i.bac.te.ra’les. N.L. masc. n. Procaibacter, a *Candidatus* genus name; *-ales*, ending to denote an order; N.L. fem. pl. n. Procaibacterales, the *Candidatus* Procaibacter order)	Procabacter; Procaibacter Horn *et al*. 2002 [43]	Garrity *et al.* 2003 [44]	D!
Saccharimonadales (=Saccharimonales)	We propose correcting the name to Saccharimonadales (Sac.cha.ri.mo.na.da’les. N.L. fem. n. Saccharimonas, a *Candidatus* genus name; *-ales*, ending to denote an order; N.L. fem. pl. n. Saccharimonadales, the *Candidatus* Saccharimonas order)	Saccharimonas Albertsen *et al*. 2013 [45]	Lemos *et al.* 2019 [46]	D!
Solibacterales	So.li.bac.te.ra’les. N.L. masc. n. Solibacter, a *Candidatus* genus name; *-ales*, ending to denote an order; N.L. fem. pl. n. Solibacterales, the *Candidatus* Solibacter order	Solibacter Sabree *et al*. 2006 [47]	Brons and van Elsas 2008 [48]	D!
Sulfidibacteriales	Sul.fi.di.bac.te.ri.a’les. N.L. neut. n. Sulfidibacterium, a *Candidatus* genus name; *-ales*, ending to denote an order; N.L. fem. pl. n. Sulfidibacteriales, the *Candidatus* Sulfidibacterium order	Sulfidibacterium Leng *et al*. 2022 [49]	Leng *et al.* 2022 [49]	D!
Thermoprofundales	Ther.mo.pro.fun.da’les. N.L. masc. n. Thermoprofundus, a yet-to-be-proposed name for the type genus; *-ales*, ending to denote an order; N.L. fem. pl. n. Thermoprofundales, the *Candidatus* Thermoprofundus order		Zhou *et al.* 2019 [50]	D!; T!
Thetyobacterales (=Tethybacterales)	We propose correcting the name to Thetyobacterales (The.ty.o.bac.te.ra’les. N.L. masc. n. Tethyobacter, a *Candidatus* genus name; *-ales*, ending to denote an order; N.L. fem. pl. n. Thetyobacterales, the *Candidatus* Tethyobacter order)	Tethyobacter corrig. Taylor *et al*. 2021=Tethybacter (sic) Taylor *et al.* 2021 [51]	Taylor *et al.* 2021 [51]	D!
Troglogloeales	Tro.glo.gloe.a’les. N.L. fem. n. Troglogloea, a *Candidatus* genus name; *-ales*, ending to denote an order; N.L. fem. pl. n. Troglogloeales, the *Candidatus* Troglogloea order	Troglogloea Kostanjšek *et al*. 2013 [52]	Yu *et al.* 2022 [53]	D!

1A deviating published name, if any, is given in parentheses. See also column 2.

## Appendix 8. Other names at the rank of family

List of *Candidatus* family names proposed up to 2022 that have not yet been included in a *Candidatus* list. These names are not considered to be pro-validly published by virtue of their inclusion in this publication. Comments indicate why we doubt that they can become pro-validly published based on the given effective publication. The names are listed here solely to provide such comments, to alert readers to the existence of these names and to provide spelling corrections. Abbreviations used are as follows: D!, no description given (at least not in main document), including incidental mentions (without actually proposing the name); E!, no etymology provided; F!, name not formed from name of nomenclatural type; H!, type is a homonym of a validly published name; S!, INSDC nt accessions not found for the type species of the type genus, or description of type genus provided in supplement; and T!, no nomenclatural type designated (at least not in main document).

**Table:** 

Proposed name of the ***Candidatus*** taxon^**1**^	Etymology	Nomenclatural type	Reference	Comment
Aminicenantaceae	A.mi.ni.ce.nan.ta.ce’ae. N.L. masc. n. Aminicenans, a *Candidatus* genus name; *-aceae*, ending to denote a family; N.L. fem. pl. n. Aminicenantaceae, the *Candidatus* Aminicenans family	Aminicenans Rinke *et al*. 2013 [19]	Kadnikov *et al.* 2019 [20]	D!
Amoebophilaceae	A.mo.e.bo.phi.la.ce’ae. N.L. masc. n. Amoebophilus, a *Candidatus* genus name; *-aceae*, ending to denote a family; N.L. fem. pl. n. Amoebophilaceae, the *Candidatus* Amoebophilus family	Amoebophilus Horn *et al*. 2001 [54]	Santos-Garcia *et al.* 2014 [55]	D!
Anaerobicaceae	A.nae.ro.bi.ca.ce’ae. N.L. fem. n. Anaerobicum, a yet-to-be-proposed name for the type genus; *-aceae*, ending to denote a family; N.L. fem. pl. n. Anaerobicaceae, the *Candidatus* Anaerobicum family	Coldseepensis Zhang *et al*. 2022 [56]	Zhang *et al.* 2022 [56]	F!
Babelaceae (=Babeliaceae)	We propose correcting the name to Babelaceae (Ba.be.la.ce’ae. N.L. fem. n. Babela, a *Candidatus* genus name; *-aceae*, ending to denote a family; N.L. fem. pl. n. Babelaceae, the *Candidatus* Babela family)	Babela Cohen *et al*. 2011 [21]	Yeoh *et al.* 2016 [22]	D!
Bathanammoxibiaceae (=Bathyanammoxibiaceae)	We propose correcting the name to Bathanammoxibiaceae (Bath.an.amm.o.xi.bi.a.ce’ae. N.L. masc. n. Bathanammoxibius, a *Candidatus* genus name; *-aceae*, ending to denote a family; N.L. fem. pl. n. Bathanammoxibiaceae, the *Candidatus* Bathanammoxibius family)	Bathyanammoxibius Zhao *et al*. 2022 [57]	Zhao *et al*. 2022 [57]	D!
Biekeibacteriaceae	Bie.ke.i.bac.te.ri.a.ce’ae. N.L. neut. n. Biekeibacterium, a *Candidatus* genus name; *-aceae*, ending to denote a family; N.L. fem. pl. n. Biekeibacteriaceae, the *Candidatus* Biekeibacterium family	Biekeibacterium Davila-Santiago *et al*. 2022 [58]	Davila-Santiago *et al.* 2022 [58]	S!
Comantemaeaceae (=Comantemaea)	We propose correcting the name to Comantemaeaceae (Co.man.te.mae.a.ce’ae. N.L. fem. n. Comantemaea, a yet-to-be-proposed *Candidatus* genus name; *-aceae*, ending to denote a family; N.L. fem. pl. n. Comantemaeaceae, the *Candidatus* Comantemaea family)	Borkfalki Hildebrand *et al*. 2019 [23]	Hildebrand *et al.* 2019 [23]	F!; synonym of *Candidatus* Borkfalkiaceae Hildebrand *et al*. 2020
Caldatribacteriaceae	Cald.a.tri.bac.te.ri.a.ce’ae. N.L. neut. n. Caldatribacterium, a *Candidatus* genus name; *-aceae*, ending to denote a family; N.L. fem. pl. n. Caldatribacteriaceae, the *Candidatus* Caldatribacterium family	Caldatribacterium Dodsworth *et al*. 2013 [59]	Murray *et al.* 2020 [60]	D!
Cenarchaeaceae	Cen.ar.chae.a.ce’ae. N.L. neut. n. Cenarchaeum, a *Candidatus* genus name; *-aceae*, ending to denote a family; N.L. fem. pl. n. Cenarchaeaceae, the *Candidatus* Cenarchaeum family	Cenarchaeum DeLong and Preston 1996 [24]	DeLong and Preston 1996 [24]	E!
Chromulinivoracaceae (=Chromulinavoraceae=Chromulinivoraceae)	We propose correcting the name to Chromulinivoracaceae (Chro.mu.li.ni.vo.ra.ca.ce’ae. N.L. masc. n. Chromulinivorax, a *Candidatus* genus name; *-aceae*, ending to denote a family; N.L. fem. pl. n. Chromulinivoracaceae, the *Candidatus* Chromulinivorax family)	Chromulinivorax corrig. Deeg *et al*. 2019=Chromulinavorax (sic) Deeg *et al*. 2019 [61]	Deeg *et al.* 2019 [61]	D!
Cloacimonadaceae	Clo.a.ci.mo.na.da.ce’ae. N.L. fem. n. Cloacimonas, a *Candidatus* genus name; *-aceae*, ending to denote a family; N.L. fem. pl. n. Cloacimonadaceae, the *Candidatus* Cloacimonas family	Cloacimonas Pelletier *et al*. 2008 [62]	Dyksma and Gallert 2019 [63]	D!
Deferrimicrobiaceae	De.fer.ri.mi.cro.bi.a.ce’ae. N.L. neut. n. Deferrimicrobium, a *Candidatus* genus name; *-aceae*, ending to denote a family; N.L. fem. pl. n. Deferrimicrobiaceae, the *Candidatus* Deferrimicrobium family	Deferrimicrobium Begmatov *et al*. 2022 [25]	Begmatov *et al.* 2022 [25]	D!
Eudoromicrobiaceae (=Eudoremicrobiaceae)	We propose correcting the name to Eudoromicrobiaceae (Eu.do.ro.mi.cro.bi.a.ce’ae. N.L. neut. n. Eudoromicrobium, a *Candidatus* genus name; *-aceae*, ending to denote a family; N.L. fem. pl. n. Eudoromicrobiaceae, the *Candidatus* Eudoromicrobium family)	Eudoremicrobium Paoli *et al*. 2022 [64]	Paoli *et al.* 2022 [64]	D!
Gastranaerophilaceae	Gastr.an.ae.ro.phi.la.ce’ae. N.L. masc. n. Gastranaerophilus, a *Candidatus* genus name; *-aceae*, ending to denote a family; N.L. fem. pl. n. Gastranaerophilaceae, the *Candidatus* Gastranaerophilus family	Gastranaerophilus Soo *et al*. 2014 [42]	Gilroy *et al.* 2021 [65]	D!
Hadarchaeaceae	Had.ar.chae.a.ce’ae. N.L. neut. n. Hadarchaeum, a *Candidatus* genus name; *-aceae*, ending to denote a family; N.L. fem. pl. n. Hadarchaeaceae, the *Candidatus* Hadarchaeum family	Hadarchaeum Chuvochina *et al*. 2019 [26]	Chuvochina *et al.* 2019 [26]	D!
Hadesarchaeaceae (=Hadesarchaeacaceae)	We propose correcting the name to Hadesarchaeaceae (Ha.des.ar.chae.a.ce’ae. N.L. neut. n. Hadesarchaeum, a *Candidatus* genus name; *-aceae*, ending to denote a family; N.L. fem. pl. n Hadesarchaeaceae, the *Candidatus* Hadesarchaeum family)	Hadesarchaeum Hua *et al*. 2019 [27]	Hua *et al.* 2019 [27]	D!
Haloplasmataceae	Ha.lo.plas.ma.ta.ce’ae. N.L. neut. n. Haloplasma, a *Candidatus* genus name; *-aceae*, ending to denote a family; N.L. fem. pl. n. Haloplasmataceae, the *Candidatus* Haloplasma family	Haloplasma Zhou *et al*. 2022 [28] (*non Haloplasma* Antunes *et al*. 2008 [29])	Zhou *et al.* 2022 [28]	D!; H!
Heimdallarchaeaceae	Heim.dall.ar.chae.a.ce’ae. N.L. neut. n. Heimdallarchaeum, a *Candidatus* genus name; *-aceae*, ending to denote a family; N.L. fem. pl. n. Heimdallarchaeaceae, the *Candidatus* Heimdallarchaeum family	Heimdallarchaeum Spang *et al*. 2019 [30]	Wu *et al.* 2022 [31]	D!
Hydrothermaceae	Hy.dro.ther.ma.ce’ae. N.L. masc. n. Hydrothermus, a *Candidatus* genus name; *-aceae*, ending to denote a family; N.L. fem. pl. n. Hydrothermaceae, the *Candidatus* Hydrothermus family	Hydrothermus Chuvochina *et al*. 2019 [26]	Chuvochina *et al.* 2019 [26]	D!
Hydrothermarchaeaceae	Hy.dro.therm.ar.chae.a.ce’ae. N.L. neut. n. Hydrothermarchaeum, a *Candidatus* genus name; *-aceae*, ending to denote a family; N.L. fem. pl. n. Hydrothermarchaeaceae, the *Candidatus* Hydrothermarchaeum family	Hydrothermarchaeum Chuvochina *et al*. 2019 [26]	Chuvochina *et al.* 2019 [26]	D!
Izemoplasmataceae (=Izemoplasmaceae)	We propose correcting the name to Izemoplasmataceae (I.ze.mo.pla.sma.ta.ce’ae. N.L. fem. n. Izemoplasma, a *Candidatus* genus name; *-aceae*, ending to denote a family; N.L. fem. pl. n. Izemoplasmataceae, the *Candidatus* Izemoplasma family)	Izemoplasma Skennerton *et al*. 2016 [32]	Zheng *et al.* 2021 [33]	D!
Manganitrophaceae	Man.ga.ni.tro.pha.ce’ae. N.L. masc. n. Manganitrophus, a *Candidatus* genus name; *-aceae*, ending to denote a family; N.L. fem. pl. n. Manganitrophaceae, the *Candidatus* Manganitrophus family	Manganitrophus Yu and Leadbetter 2020 [66]	Yu *et al.* 2022 [53]	D!
Methanohydrogenotrophicaceae	Me.tha.no.hy.dro.ge.no.tro.phi.ca.ce’ae. N.L. neut. n. Methanohydrogenotrophicum, a *Candidatus* genus name; *-aceae*, ending to denote a family; N.L. fem. pl. n. Methanohydrogenotrophicaceae, the *Candidatus* Methanohydrogenotrophicum family	Methanohydrogenotrophicum Hua *et al*. 2019 [27]	Hua *et al.* 2019 [27]	D!
Methanomethylophilaceae	Me.tha.no.me.thy.lo.phi.la.ce’ae. N.L. masc. n. Methanomethylophilus, a *Candidatus* genus name; *-aceae*, ending to denote a family; N.L. fem. pl. n. Methanomethylophilaceae, the *Candidatus* Methanomethylophilus family	Methanomethylophilus Borrel *et al*. 2012 [67]	Gaci *et al.* 2014 [68]	D!
Methanomixtitrophicaceae (=Methanomixtatrophicaceae)	We propose correcting the name to Methanomixtitrophicaceae (Me.tha.no.mix.ti.tro.phi.ca.ce’ae. N.L. neut. n. Methanomixtitrophicum, a *Candidatus* genus name; *-aceae*, ending to denote a family; N.L. fem. pl. n. Methanomixtitrophicaceae, the *Candidatus* Methanomixtitrophicum family)	Methanomixtatrophicum Hua *et al*. 2019 [27]	Hua *et al.* 2019 [27]	D!
Methanoproducendaceae	Me.tha.no.pro.du.cen.da.ce’ae. N.L. neut. n. Methanoproducendum, a *Candidatus* genus name; *-aceae*, ending to denote a family; N.L. fem. pl. n. Methanoproducendaceae, the *Candidatus* Methanoproducendum family	Methanoproducendum Hua *et al*. 2019 [27]	Hua *et al.* 2019 [27]	D!
Methylacidiphilaceae	Me.thyl.a.ci.di.phi.la.ce’ae. N.L. neut. n. Methylacidiphilum, type genus of the family; suff. *-aceae*, ending to denote a family; N.L. fem. pl. n. Methylacidiphilaceae, the family Methylacidiphilum	Methylacidiphilum Hou *et al*. 2008 [36]	Op den Camp *et al.* 2009 [37]	D!
Methylarchaeaceae	Me.thyl.ar.chae.a.ce’ae. N.L. neut. n. Methylarchaeum, a *Candidatus* genus name; *-aceae*, ending to denote a family; N.L. fem. pl. n. Methylarchaeaceae, the *Candidatus* Methylarchaeum family	Methylarchaeum Hua *et al*. 2019 [27]	Ou *et al.* 2022 [38]	D!
Microvillispirillaceae	Mi.cro.vil.li.spi.ril.la.ce’ae. N.L. neut. n. Microvillispirillum, a yet-to-be-proposed *Candidatus* genus name; *-aceae*, ending to denote a family; N.L. fem. pl. n. Microvillispirillaceae, the *Candidatus* Microvillispirillum family	Rimicarispirillum Aubé *et al*. 2022 [69]	Aubé *et al.* 2022 [69]	D!
Monstrimarinaceae (=Monstramariaceae)	We propose correcting the name to Monstrimarinaceae (Mon.stri.ma.ri.na.ce’ae. N.L. fem. n. Monstrimarina, a yet-to-be-proposed *Candidatus* genus name; *-aceae*, ending to denote a family; N.L. fem. pl. n. Monstrimarinaceae, the *Candidatus* Monstrimarina family)		Landry *et al.* 2017 [39]	D!; T!
Nanohydrothermaceae	Na.no.hy.dro.ther.ma.ce’ae. N.L. masc. n. Nanohydrothermus, a *Candidatus* genus name; L. fem. pl. suff. *-aceae*, ending to denote a family; N.L. fem. pl. n. Nanohydrothermaceae, the *Candidatus* Nanohydrothermus family	Nanohydrothermus Xie *et al*. 2022 [41]	Xie *et al.* 2022 [41]	D!; S!
Nanopusillaceae	Na.no.pu.sil.la.ce’ae. N.L. masc. n. Nanopusillus, a *Candidatus* genus name; *-aceae*, ending to denote a family; N.L. fem. pl. n. Nanopusillaceae, the *Candidatus* Nanopusillus family	Nanopusillus Wurch *et al*. 2016 [70]	Huber *et al.* 2016 [71]	D!
Nanosalenecaceae (=Nanosalenecusaceae)	We propose correcting the name to Nanosalenecaceae (Na.no.sal.en.e.ca.ce’ae. N.L. masc. n. Nanosalenecus, a *Candidatus* genus name; *-aceae*, ending to denote a family; N.L. fem. pl. n. Nanosalenecaceae, the *Candidatus* Nanosalenecus family)	Nanosalenecus Xie *et al*. 2022 [41]	Xie *et al.* 2022 [41]	D!; S!
Nucleotidisoteraceae	Nu.cle.o.ti.di.so.ter.a.ce’ae N.L. masc. n. Nucleotidisoter, a *Candidatus* genus name; L. fem. pl. suff. *-aceae*, ending to denote a family; N.L. fem. pl. n. Nucleotidisoteraceae, the *Candidatus* Nucleotidisoter family	Nucleotidisoter Xie *et al*. 2022 [41]	Xie *et al.* 2022 [41]	D!; S!
Nucleotidivindicaceae	Nu.cle.o.ti.di.vin.di.ca.ce’ae N.L. masc. n. Nucleotidivindex, a *Candidatus* genus name; L. fem. pl. suff. *-aceae*, ending to denote a family; N.L. fem. pl. n. Nucleotidivindicaceae, The *Candidatus* Nucleotidivindex family	Nucleotidivindex Xie *et al*. 2022 [41]	Xie *et al.* 2022 [41]	D!; S!
Perseobacteraceae (=Persebacteraceae)	We propose correcting the name to Perseobacteraceae (Per.se.o.bac.te.ra.ce’ae. N.L. masc. n. Perseobacter, a *Candidatus* genus name; *-aceae*, ending to denote a family; N.L. fem. pl. n. Perseobacteraceae, the *Candidatus* Perseobacter family)	Persebacter Taylor *et al*. 2021 [51]	Taylor *et al.* 2021 [51]	D!
Polydorobacteraceae (=Polydorabacteraceae)	We propose correcting the name to Polydorobacteraceae (Po.ly.do.ro.bac.te.ra.ce’ae. N.L. masc. n. Polydorobacter, a yet-to-be-proposed *Candidatus* genus name; *-aceae*, ending to denote a family; N.L. fem. pl. n. Polydorobacteraceae, the *Candidatus* Polydorobacter family)		Waterworth *et al.* 2021 [72]	D!; T!
Procaibacteraceae (=Procabacteraceae)	We propose correcting the name to Procaibacteraceae (Pro.ca.i.bac.ter.a.ce’ae. N.L. masc. n. Procaibacter, a *Candidatus* genus name; *-aceae*, ending to denote a family; N.L. fem. pl. n. Procaibacteraceae, the *Candidatus* Procaibacter family)	Procaibacter corrig. Horn *et al.* 2002 = Procabacter (sic) Horn *et al.* 2002 [43]	Garrity *et al.* 2003 [44]	D!
Rhabdochlamydiaceae	Rhab.do.chla.my.di.a.ce’ae. N.L. fem. n. Rhabdochlamydia, a *Candidatus* genus name; *-aceae*, ending to denote a family; N.L. fem. pl. n. Rhabdochlamydiaceae, the *Candidatus* Rhabdochlamydia family	Rhabdochlamydia Kostanjšek *et al*. 2004 [73]	Horn 2010 [74]	[pro-validate? type PVP]
Roseilineaceae	Ro.se.i.li.ne.a.ce’ae. N.L. fem. n. Roseilinea, a *Candidatus* genus name; *-aceae*, ending to denote a family; N.L. fem. pl. n. Roseilineaceae, the *Candidatus* Roseilinea family	Roseilinea Thiel *et al*. 2016 [75]	Ward *et al.* 2021 [76]	D!
Saccharimonadaceae	Sac.cha.ri.mo.na.da.ce’ae. N.L. fem. n. Saccharimonas, a *Candidatus* genus name; *-aceae*, ending to denote a family; N.L. fem. pl. n. Saccharimonadaceae, the *Candidatus* Saccharimonas family	Saccharimonas Albertsen *et al*. 2013 [45]	Lemos *et al.* 2019 [46]	D!
Tethyobacteraceae (=Tethybacteraceae)	We propose correcting the name to Tethyobacteraceae (Te.thy.o.bac.te.ra.ce’ae. N.L. masc. n. Tethyobacter, a *Candidatus* genus name; *-aceae*, ending to denote a family; N.L. fem. pl. n. Tethyobacteraceae, the *Candidatus* Tethyobacter family)	Tethyobacter corrig. Taylor *et al.* 2021 = Tethybacter (sic) Taylor *et al.* 2021 [51]	Taylor *et al.* 2021 [51]	D!
Thermochlorobacteraceae (=Thermochlorobacteriaceae)	We propose correcting the name to Thermochlorobacteraceae (Ther.mo.chlo.ro.bac.te.ra.ce’ae. N.L. masc. n. Thermochlorobacter, a *Candidatus* genus name; *-aceae*, ending to denote a family; N.L. fem. pl. n. Thermochlorobacteraceae, the *Candidatus* Thermochlorobacter family)	Thermochlorobacter Liu *et al*. 2012 [77]	Liu *et al.* 2012 [77]	D!
Veterimethanophagaceae (=Veteromethanophagaceae)	We propose correcting the name to Veterimethanophagaceae (Ve.te.ri.me.tha.no.pha.ga.ce’ae. N.L. masc. n. Veterimethanophagus, a yet-to-be-proposed *Candidatus* genus name; *-aceae*, ending to denote a family; N.L. fem. pl. Veterimethanophagaceae, the *Candidatus* Veterimethanophagus family)		Vulcano *et al.* 2022 [78]	D!; T!

1A deviating published name, if any, is given in parentheses. See also column 2.

## Abbreviations

ICNP: International Code of Nomenclature of Prokaryotes
